# Innovative Design and Development of Personalized Ankle-Foot Orthoses for Survivors of Stroke With Equinovarus Foot: Protocol for a Feasibility and Comparative Trial

**DOI:** 10.2196/52365

**Published:** 2024-04-02

**Authors:** Rui Silva, Pedro Morouço, Jorge Lains, Paula Amorim, Nuno Alves, António Prieto Veloso

**Affiliations:** 1 Centre for Rapid and Sustainable Product Development Polytechnic University of Leiria Marinha Grande Portugal; 2 CIPER Faculdade de Motricidade Humana Lisboa Portugal; 3 Escola Superior de Educação e Ciências Sociais Polytechnic University of Leiria Leiria Portugal; 4 Centro de Medicina de Reabilitação da Região Centro–Rovisco Pais Tocha Portugal; 5 Faculty of Medicine University Coimbra Coimbra Portugal

**Keywords:** 3D printing, 3D scanner, ankle foot orthosis, biomechanical analysis, equinovarus foot

## Abstract

**Background:**

Ankle-foot orthoses (AFOs) are vital in gait rehabilitation for patients with stroke. However, many conventional AFO designs may not offer the required precision for optimized patient outcomes. With the advent of 3D scanning and printing technology, there is potential for more individualized AFO solutions, aiming to enhance the rehabilitative process.

**Objective:**

This nonrandomized trial seeks to introduce and validate a novel system for AFO design tailored to patients with stroke. By leveraging the capabilities of 3D scanning and bespoke software solutions, the aim is to produce orthoses that might surpass conventional designs in terms of biomechanical effectiveness and patient satisfaction.

**Methods:**

A distinctive 3D scanner, complemented by specialized software, will be developed to accurately capture the biomechanical data of leg movements during gait in patients with stroke. The acquired data will subsequently guide the creation of patient-specific AFO designs. These personalized orthoses will be provided to participants, and their efficacy will be compared with traditional AFO models. The qualitative dimensions of this experience will be evaluated using the Quebec User Evaluation of Satisfaction With Assistive Technology (QUEST) assessment tool. Feedback from health care professionals and the participants will be considered throughout the trial to ensure a rounded understanding of the system’s implications.

**Results:**

Spatial-temporal parameters will be statistically compared using paired *t* tests to determine significant differences between walking with the personalized orthosis, the existing orthosis, and barefoot conditions. Significant differences will be identified based on *P* values, with *P*<.05 indicating statistical significance. The Statistical Parametric Mapping method will be applied to graphically compare kinematic and kinetic data across the entire gait cycle. QUEST responses will undergo statistical analysis to evaluate patient satisfaction, with scores ranging from 1 (not satisfied) to 5 (very satisfied). Satisfaction scores will be presented as mean and SD values. Significant variations in satisfaction levels between the personalized and existing orthosis will be assessed using a Wilcoxon signed rank test. The anticipation is that the AFOs crafted through this innovative system will either match or outperform existing orthoses in use, with higher patient satisfaction rates.

**Conclusions:**

Embracing the synergy of technology and biomechanics may hold the key to revolutionizing orthotic design, with the potential to set new standards in patient-centered orthotic solutions. However, as with all innovations, a balanced approach, considering both the technological possibilities and individual patient needs, will be paramount to achieving optimal outcomes.

**International Registered Report Identifier (IRRID):**

PRR1-10.2196/52365

## Introduction

### Overview

Stroke, often termed a cerebrovascular accident, poses a monumental global health issue and stands as the second leading cause of mortality worldwide [1]. In addition to the grave concern of mortality, survivors of stroke frequently grapple with substantial morbidity, most notably neurological impairments that substantially hamper their quality of life. Among these impairments, a prevalent issue is equinovarus foot, a symptom characterized by the foot being plantarflexed (downward) and inverted (turned inward), often resulting from muscle imbalances or neurological impairments [2,3]

In the management and rehabilitation of the equinovarus foot, ankle-foot orthoses (AFOs) serve as a foundational element, supporting and aligning the ankle and foot, suppressing spastic and overpowering muscles, and assisting weak or paralyzed muscles [4]. While these devices are indispensable in aiding patients to regain some semblance of normal gait, they come with their own sets of limitations. Broadly speaking, AFOs are categorized into 2 primary types: traditional off-the-shelf models and custom-crafted versions. Traditional AFOs, designed for a broad patient demographic, offer widespread accessibility but often miss the mark in addressing the unique biomechanical needs of each patient. This one-size-fits-all approach has drawn criticism for its rigidity and lack of individual customization [5]. Conversely, custom-made AFOs are meticulously tailored to fit a specific patient’s anatomical structure. While they provide a more individualized fit, the process of creating these orthoses is time-consuming and very laborious. In addition, the process is also wasteful of materials, as plaster molds and other excess fabrication materials are discarded during the fabrication process [6]. This gap between age-old craftsmanship and cutting-edge precision sets the stage for technological intervention, aiming to meld the advantages of both approaches.

The concept of reverse engineering in orthotics involves capturing a patient’s limb anatomy in great detail, translating this information into a digital model, and then crafting an orthotic device to perfectly align with the individual’s biomechanical demands [7,8]. Using 3D scanning techniques allows for a highly accurate representation of human anatomy. This digital replica serves as a blueprint upon which orthotic devices can be meticulously designed, thereby ensuring that the device is tailored to an individual’s unique biomechanical requirements. Nevertheless, the integration of 3D scanning technology into the orthotic field is fraught with challenges. Capturing a comprehensive scan, particularly of the plantar region of the foot, proves to be problematic. The quality of the scan is often compromised due to patient movements, exacerbated by the extended duration needed for the scanning process [9]. This prolonged duration can be uncomfortable for the patient, thereby leading to unintended movements and consequential errors in the scan data. Moreover, there are ongoing debates over the computational workload and adaptability of the resulting digital models. Such pitfalls, whether arising from anatomical complexities, patient movements, or technological limitations, could culminate in an improperly fitting orthotic device.

The science of photogrammetry, which involves making measurements based on photographs, offers a potential solution. Initially used for mapping and topographical studies [10], its application in the medical realm, particularly in orthotics and prosthetics, has only recently been explored. The capacity to transform photographs into intricate 3D models offers quicker scan times and could minimize errors induced by patient movements [11]. However, the full-scale integration of this promising technology into the orthopedic field is still in its infancy [12-16]. Ensuring that the resulting 3D models are an accurate reflection of patient anatomy and that the resultant devices are both functional and comfortable remains a challenge. Furthermore, orthopedics is a multidisciplinary field that includes physicians, physical therapists, and engineers. Consequently, any new technological adoption must be orchestrated carefully to ensure effective use across all these professions [17]. Armed with these technological advancements, the field of orthotics is poised for a transformative evolution—a shift toward a more patient-centric and technologically integrated paradigm. This fusion of traditional orthotic craftsmanship with cutting-edge computational tools heralds a new era in patient care, targeting both precision and broad accessibility.

### Goal of This Study

This research protocol delineates our approach to developing a next-generation AFO system tailored to meet the specific needs of survivors of stroke. The primary objective is to harness advanced scanning tools and bespoke software for a holistic orthotic solution. By innovatively integrating technology and medical expertise, we envision a transformation in the rehabilitation journey, creating a more refined and effective recovery pathway for individuals with poststroke motor challenges. Our methodological framework will guide us from the initial stages of scanner and software development to a culminating phase of validation, where the proposed orthotic devices will undergo rigorous patient trials. Through this initiative, we aim to chart a progressive path in the realm of poststroke orthotic care.

## Methods

### Study Design

This nonrandomized feasibility study aims to harness advanced scanning technologies and innovative software for the design and refinement of orthotics tailored specifically to the unique anatomical and biomechanical needs of survivors of stroke presenting with equinovarus deformity. Following a noninferiority trial design for biomechanical outcomes and a superiority trial design for qualitative outcomes, our methodology focuses on the development of a novel AFO system. The goal is to ensure its biomechanical performance is at least as effective as off-the-shelf AFOs while also enhancing patient satisfaction. Feedback from patients and clinical observations will serve as the primary indicators of success.

### Ethical Considerations

The approval for the protocol of this study was granted by the Health Ethics Committee of the Centro de Medicina de Reabilitação da Região Centro–Rovisco Pais (Tocha, Portugal) in August 2022.

### Consent to Participate and Consent for Publication

#### Overview

A document was developed at the request of the health ethics committee for informed, clear, and voluntary consent for participation in research studies. The document outlines the research study’s objective and assures that there will be no detriment to treatments and clinical follow-up should the patient choose to withdraw. It also guarantees the anonymity and confidentiality of all collected data, including photographs, results from the Quebec User Evaluation of Satisfaction With Assistive Technology (QUEST) [18], and biomechanical analysis data. The consent form must be signed by both the attending physician and the patient.

This protocol was prepared according to the SPIRIT (Standard Protocol Items: Recommendations for Interventional Trials) 2013 checklist for reporting a protocol study [19].

#### Eligibility Criteria and Recruitment Procedures

The inclusion criteria for this study have been defined with precision to select the most suitable candidates in alignment with the study objectives. We are targeting survivors of stroke, both male and female, aged between 18 and 75 years, who exhibit equinovarus foot secondary to hemiparesis, affecting either the left or right side. A prerequisite for potential participants is their current use of AFOs. Furthermore, the concurrent use of any assistive technologies such as tripods, crutches, or canes is deemed acceptable. Essential criteria include the capacity to provide informed consent and the ability to ambulate, either independently or with the support of the aforementioned devices. Conversely, candidates with concomitant neurological or orthopedic conditions that might confound the study outcomes, those with active dermatological conditions, or those with severe communication impairments potentially hindering consistent participation will be excluded.

The recruitment process will be at the Centro de Medicina de Reabilitação da Região Centro. Attending physicians will review patient profiles to identify individuals meeting the stipulated criteria. Those aligning with our research parameters will be briefed on the study’s aims and subsequently provided with a detailed consent document. Upon granting written consent, these individuals will be enlisted as participants, ensuring a systematic and ethically rigorous approach to data acquisition and feedback.

#### Clinical Outcomes

In the pursuit of developing an optimized orthotic design system, an array of clinical metrics is implemented to gauge its efficiency, efficacy, and the comfort it bestows on both patients and health care professionals. Ensuring a comfortable experience for the patient during the photography process is paramount, given its pivotal role in orthotic design. This precision not only benefits the patient but also ensures that the system health care professionals navigate is intuitive.

Biomechanical assessments use the Qualisys Miqus M3 system, paired with Bertec force platforms. Patients will wear the Calibrated Anatomical System Technique lower body marker set, which consists of 36 reflective markers, as prescribed by Cappozzo et al [20]. Observations cover 3 walking conditions for each participant: unaided (where possible), with the current orthosis, and with the newly designed orthosis. This methodology provides an in-depth understanding of the orthosis’s efficacy, drawing from 10 walking cycles for each leg, and analyzing both kinematics and kinetics.

The biomechanical data under scrutiny spans temporal-spatial parameters, which capture walking speed, gait cycle duration, step length, step time, time in stance, and time in swing. Kinematic parameters delve into pelvic movements such as anterior tilt, up obliquity, and internal rotation. Hip parameters include flexion, adduction, and internal rotation, while knee parameters assess flexion, varus, and internal rotation. Ankle and foot evaluations note dorsiflexion, inversion, pitch, and internal progression. Kinetic parameters are marked by the internal moments at the hip (extensor and valgus), knee (extensor and valgus), and ankle (plantarflexor and extensor), accompanied by the vertical ground reaction force.

The qualitative patient analysis will also incorporate the QUEST assessment. QUEST focuses on understanding the user’s satisfaction with assistive technology. It evaluates a range of aspects, from device functionality to user confidence. This offers insights into patients’ perceptions and benefits derived from the new orthosis in comparison to conventional models. Incorporating QUEST ensures the orthosis not only meets clinical requirements but also aligns with patient preferences and comfort levels.

Through these comprehensive evaluations, the study aims to offer an enriched perspective on the potential and effectiveness of the innovative orthotic system.

### Data Analysis

The forthcoming data analysis is designed to provide an in-depth understanding of the impact of personalized orthoses on gait parameters in relation to both preexisting orthosis and barefoot walking. The sample size was estimated at a prespecified power of 90%, while the α value was set at <.05. The primary outcomes will be represented through spatial-temporal data tables and normalized gait graphs, spanning from 0% to 100% of the gait cycle for the left and right legs.

Spatial-temporal parameters will undergo statistical comparisons using paired *t* tests. This will discern any significant differences between walking with the personalized orthosis, the preexisting orthosis, and walking barefoot. Significant distinctions will be recognized based on *P* values, with a threshold set at 95% indicating statistical significance.

Graphical comparisons of kinematic and kinetic data will use the Statistical Parametric Mapping (SPM) method. SPM is tailored for the analysis of 1D biomechanical data series, such as kinematic curves, yielding a nuanced understanding of differences across the entire gait cycle rather than mere isolated time points. The analysis will leverage the *SPM1D* script. By using SPM1D, it becomes feasible to pinpoint regions in the gait cycle where palpable differences between conditions (existing orthosis, personalized orthosis, and barefoot) arise. This rigorous method offers a continuous evaluation over the entire time or space continuum, safeguarding against missing subtle yet clinically pivotal variations.

Simultaneously, the QUEST responses will be statistically analyzed to evaluate patient satisfaction. Scores from the QUEST, which range from 1 (not satisfied) to 5 (very satisfied), will be presented as mean and SD values for each question. A 1-sample *t* test will be used to determine if the mean satisfaction scores significantly differ from a neutral value. Additionally, a Wilcoxon signed rank test may be used to determine differences in satisfaction levels between using the personalized orthosis and the preexisting orthosis. Any statistically significant variations in user satisfaction between the 2 orthoses will provide insight into the preferential use and comfort of the personalized design.

In essence, this multifaceted statistical approach aims to quantify not only the possible biomechanical advantages of personalized orthoses over standard ones but also the subjective satisfaction of users, ensuring a holistic assessment of the new system’s efficacy.

## Results

The methodology and approach of this research harbor specific expectations concerning its outcomes. We will use the Qualisys Track Manager from Qualisys to capture biomechanical data with unparalleled accuracy. Once gathered, the data will be processed and analyzed rigorously. With the integration of the Project Automation Framework from Qualisys and Visual 3D from C-Motion, the raw biomechanical data will be transformed into actionable insights that promise to inform and refine orthotic design.

One of the primary quantitative expectations is that the orthosis developed through the new system will either match or surpass the performance of the patient’s current orthosis. This benchmark stems from the belief that the integration of state-of-the-art technology and personalized biomechanical data can achieve superior orthotic design. On the qualitative front, using the QUEST assessment, the expectation leans toward higher satisfaction rates with the new orthosis. Since the orthosis is tailored specifically to the patient’s leg, it is anticipated that its unique design will resonate more with patients, ensuring better fit, comfort, and overall user experience. To ensure comprehensive results, feedback from health care professionals and participants will be actively sought throughout the trial phases. This blend of qualitative and quantitative data aims to present a holistic perspective on the impact of the new orthotic design, setting the stage for potential breakthroughs in patient-centered orthotic solutions. In summary, while this research protocol lays out the groundwork and anticipated outcomes, the subsequent study will seek to not just present numbers but to demonstrate the tangible and intangible benefits of a personalized orthotic approach.

## Discussion

Over the years, the field of gait rehabilitation has witnessed significant advancements, with orthoses taking center stage in many innovative solutions. As such, they have played a pivotal role in enhancing gait and laying the foundation for more customized interventions [21,22]. In the chronicle of medical interventions, the present times showcase a blend of time-tested traditional methods coexisting with avant-garde technologies. It is within this dynamic backdrop that the new system emerges, positioning itself as a game changer in the realm of orthoses. With a design methodology that captures the transformative essence of technology, this system aims to usher in a new epoch where AFOs are no longer generic but are sculpted based on the detailed biomechanical nuances of individual patients [23].

A key component of this innovation lies in the use of 3D scanning and 3D printing techniques. Particularly, AFOs crafted through such state-of-the-art processes have been thrust under the academic microscope. In recent years, various studies have examined multiple outcomes with the use of these technologies for the fabrication of AFOs. Belokar et al [24] and Cha et al [25] conducted numerous mechanical tests to understand the strength and deformation of the AFO, while other studies focused on gait analysis [26-28], while others on a qualitative analysis of patient comfort [16,29]. The allure of these techniques is evident, offering unparalleled precision coupled with the prospect of personalization. However, as with all innovations, there is a spectrum of opinions. While numerous research endeavors highlight the undeniable advantages of 3D methodologies, others have voiced concerns—touching upon biomechanical compatibility, the robustness of materials used, and the overall comfort on prolonged usage studies [8].

While contrasting the biomechanics of barefoot walking with orthotic-assisted gait yields valuable insights, our central focus is on the differences between traditionally designed orthoses and those created using the novel system. Contemporary research reinforces the merits of tailored medical interventions, suggesting that custom orthoses can lead to enhanced foot function, pain relief, and overall improved mobility [30,31]. For patients, the benefits of this approach are substantial. Custom-made orthoses, derived from comprehensive biomechanical analyses, not only promise greater comfort but also accelerate gait rehabilitation and minimize complications arising from poorly fitted orthoses [6,7,25]. Such initiatives are in tune with the broader shift in health care toward patient-centered treatments, ensuring holistic and efficacious therapeutic outcomes [32].

Nonetheless, potential limitations exist. While the novel system promises tailored orthoses, individual patient responses, adaptation periods, and unique rehabilitation timelines could present challenges. The variability in individual reactions to orthoses, both in terms of comfort and therapeutic outcomes, remains a critical factor to consider.

This proposed research protocol marks a pivotal juncture between technology and biomechanics in the health care landscape. It signals a shift in orthotic design, embracing recent advancements and a nuanced understanding of biomechanics. The endeavors are not merely about gait rehabilitation recovery but also about setting a new benchmark for precision and efficacy in patient care.

## Appendix

Multimedia Appendix 1 Peer review report.

## Abbreviations

AFO: ankle-foot orthosis
QUEST: Quebec User Evaluation of Satisfaction With Assistive Technology
SPIRIT: Standard Protocol Items: Recommendations for Interventional Trials
SPM: Statistical Parametric Mapping
