# Supplementary material for: Innovative Design and Development of Personalized Ankle-Foot Orthoses for Survivors of Stroke With Equinovarus Foot: Protocol for a Feasibility and Comparative Trial
Source: JMIR Res Protoc. 2024 Apr 2;13:e52365. doi: 10.2196/52365 (PMC11022130; doi:10.2196/52365)
Supplement: Multimedia Appendix 1 [file resprot_v13i1e52365_app1.pdf]

FCT - DEPARTAMENTO DE FORMAÇÃO AVANÇADA

Concurso para Atribuição de Bolsas de Doutoramento 2019

Rui Miguel Almeida Dias Silva
Referência: SFRH/BD/145292/2019
Candidatura avaliada pelo painel 'Engenharia Mecânica'

A Conceder (*)

Critérios	Factor de Ponderação	Classificação (min=1;máx=5)	SubCritérios & Observações
**Mérito do Candidato**	4	4,450	Designação / Factor de Ponderação / Classificação (min=1;máx=5)<br>Subcritério A1 (5) 4,500<br>Subcritério A2 (4) 4,500<br>Subcritério A3 (1) 4,000<br>Bonificação (grau de incapacidade) : 0%<br><br>Licenciatura em Biomecanica com 14 valores, Mestrado em Desporto e Saúde para Crianças e Jovens-19 valores. Investigador (projecto BioMATE) e Assistente convidado do IPL. 1 capitulo de livro, 3artigos em conferencia, 1 prémio de mérito. Grande motivação na continuação do trabalho desenvolvido no MsC e como colaborador no BioMATE. Muito boa carta de motivação.
**Mérito do programa de trabalhos a desenvolver**	4	4,300	O objectivo é desenvolver e optimizar um sistema de produção de ortóteses AFO costumizadas, simples e rápido, para pacientes que sofreram AVC ou PC. Interesse para a melhoria da qualidade de vida. Desenvolver um processo de fabrico das AFO por fabricação aditiva, com recurso a modelos 3D reais , obtendo formas e arquiteturas complexas, personalizadas, num curto espaço de tempo. Bibliografia adequada embora a maior parte com mais de 5 anos. Metodologia adequada e dividida em tarefas bem especificas da aquisição de imagens, produção de impressora 3D hibrida e multimaterial,cosntrução de software user-friendly, avaliação e caracterização dos produtos obtidos utilizando várias técnicas de caracterização de materiais. Apresenta cronograma e diagrama esquemático das tarefas. Questiona-se aqui se a formação do candidato é a adequada para concretizar os objectivos propostos, sendo que se acredita que, neste caso, o acompanhamento dos orientadores é especialmente crítico.
**Mérito das condições de acolhimento**	2	5,000	O orientador e co-orientadores tem experiência de orientação cientifica e de realização de trabalho de I&D, apresentando perfis e

(considerar tanto a Instituição como o Responsável pelas actividades)	formações complementares entre si,o que beneficiará o candidato. As instituições de acolhimento tem as condições necessárias para acolher o programa de trabalhos.

Classificação Final 4,500

Para o painel 'Engenharia Mecânica' as candidaturas com classificação inferior a **3,776** estão propostas para recusa.

(*) A conceder condicionalmente, desde que elegível de acordo com o Estatuto do Bolseiro e o Regulamento de Bolsas de Investigação da Fundação para a Ciência e Tecnologia, I.P.
